# A Novel Versatile Approach for Underwater Conformal Volumetric Array Design

**DOI:** 10.3390/s21113591

**Published:** 2021-05-21

**Authors:** Taofeek Ayotunde Yusuf, Seonghun Pyo, Yongrae Roh

**Affiliations:** School of Mechanical Engineering, Kyungpook National University, Daegu 41556, Korea; yustaofeekay@yahoo.com (T.A.Y.); nasgool@naver.com (S.P.)

**Keywords:** conformal volumetric array, optimization, beamwidth, ripple, FEA

## Abstract

In this study, we present a novel approach to the design of a conformal volumetric array composed of M × N convex subarrays in two orthogonal curvilinear directions for underwater acoustic imaging for mine detection. Our design targets require that the proposed array transducer has three-dimensional half-power beamwidths of 85° and 25° in either of its convex subarray parts, while also reaching a peak transmitting voltage response above 147 dB. The radiated sound pressure of the subarrays was independently derived as a function of their geometrical parameters. The resulting directional factors were then combined to analyze the beam profile of the entire array. The design was finally optimized to minimize the ripple level. To validate this theoretical design, the structure was modeled and analyzed using the finite element method. The comparison between the resulting beam pattern from the finite element analysis and the analytical computation showed an excellent compliance. The method advanced is a simple and systematic analytical model to facilitate the development of new conformal volumetric arrays for underwater mine detection.

## 1. Introduction

For decades, conformal arrays have been used in non-destructive evaluation [1], medical imaging and diagnosis [2], and underwater sound navigation and ranging (SONAR) [3]. Among these, underwater SONAR has become prominent in the contemporary use of such arrays, providing surveillance for maritime safety and security against threats such as sea mines [4,5,6,7,8]. Many recent studies are dedicated to naval mine detection, mine-like object classification, and mine countermeasures [9,10,11,12,13,14,15,16].

Conformal arrays structurally conform to their host’s surface, appearing like an integral part of the whole structure with radiating elements arranged on a curved surface [17]. Compared to a planar array, they provide a wider beamwidth, smaller profile, and reduced drag from hydrodynamic forces [18,19]. These arrays are generally known for their complex geometry, making them difficult to design and analyze [20,21]. Hence, syntheses of these arrays’ geometry are less commonly done analytically using the fundamental acoustic equations, as is typical with the case of planar arrays [22,23].

In fact, few existing designs in the literature have a mathematical background which could have facilitated more understanding of the conformal arrays [24]. The literature does not provide sufficient techniques to guide the design of a conformal array. The common approaches including the popular Dolph-Chebyshev design and the genetic algorithm have been criticized for time delay and exclusion of radiation pattern of the elements in the analysis, respectively [25]. As with the other techniques to help the design of conformal arrays, the equivalent circuit method has recently been proposed [26], which usually involves complex algorithms. The finite element method and other advanced simulation software or computer application packages are utilized as well [27,28,29,30]. An alternative design technique known as transformation optics (TO) has also been applied in new studies [31]. As an integration of mapping and coordinate transformation techniques, the major goal of TO is to find a conformal array having an equivalent performance with a linear or planar array [32]. 

In many other studies, the practical construction of these arrays is carried out by simply bending them around the curvature of a host surface [33,34,35,36]. Meanwhile, the transducer made in such a manner has been identified with several problems such as shift in resonance frequency, change in performance, and mismatch between the diameter of the transducer and the bending radius [37]. Consequently, there is a need for a more versatile design, one that is intrinsically conformable to any given shape by simple modification of the structural variables or can provide any given beamwidth depending on the performance requirement. This is only possible through the development of a characteristic equation upon which the geometrical parameters of the models are fundamentally related. 

Meanwhile, whereas there has been a quantum of research efforts [38,39,40,41,42] on cylindrical conformal arrays, the research on conformal volumetric arrays is comparatively less abundant. These few existing studies are focused on the sparse volumetric array and aim to narrow the main lobe width or the side lobe levels [43,44], while the long-range detection in acoustic imaging requires broader beamwidth [45]. Besides the wider beamwidth characteristics [46], the two curvilinear parts of the uniform volumetric array geometry would enhance a better penetration depth than the cylindrical array.

In this study, we present a novel approach to designing a 5 × 55 conformal volumetric array for mine detection considering the performance limitations in the designs. Typically, in principle, low-frequency signals favor long range while high frequency favors resolution. Meanwhile, 100 kHz is the maximum in the low ultrasonic frequency range. In our approach, this frequency was chosen to achieve a sufficient range detection at a maximum possible resolution in an underwater application [47,48]. At this frequency, the advantage of broader beamwidth offered by the doubly curved volumetric array was employed to provide a long-ranging detection [45]. The conformal array adopted also compensates for the problem of small radiation areas peculiar to some low-frequency, high-power transducers used in this application [3] while at the same time constricting the aperture width to moderate the overall size of this complex structure for economic consideration [49]. 

However, the design scheme in this work was developed as a universal model without any restrictions on the frequency it can be applied to. Hence, the scheme can be utilized to design a conformal volumetric array working at any arbitrary frequency depending on the interest of the designer. In this work, the design method is applied to the sample frequency of 100 kHz and geometry as a specific case.

The conformal volumetric array consists of two convex subarray parts that are composed of M × N rectangular elements. The sound pressure from each subarray was mathematically derived to obtain its respective directional factors. Using the product theorem, the directional factor of the whole structure was composed to characterize the beam pattern [50]. Consequently, M and N values required to achieve −3 dB beamwidths of 85° and 25° in the respective subarray part were determined through an optimization technique in addition to minimizing the ripple level to achieve a robust design. Finally, computational analysis was performed using the finite element method to validate the theoretical results as well as evaluate the transmitting voltage response (TVR) of the resulting conformal array.

The novelty of this work is in deriving a new equation to design conformal volumetric array, which enables the theoretical analysis of the array performance for the first time, to our knowledge. The uniqueness of the design equation is in the inclusion of curvature as an active determinant variable of the performance output. This is an uncommon approach to designing a conformal array due to the complex geometry of the array. This is a simpler and faster analytical approach compared to the use of complex algorithms and design software in previous works.

## 2. The Conformal Array Geometrical Parameters

**Figure 1:**
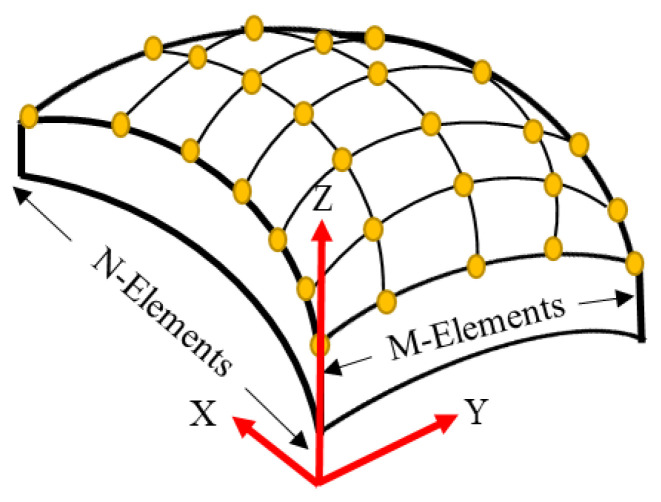
The schematic structure of the M × N volumetric array of point sources.

**Figure 2:**
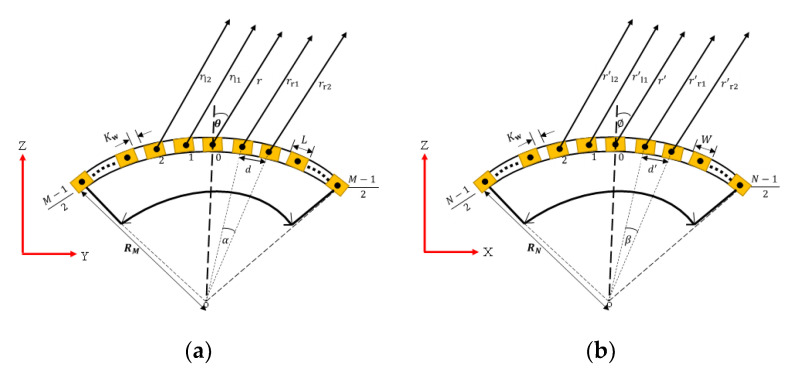
Idealized geometry of the conformal array: (**a**) M-subarray part; (**b**) N-subarray part.

Figure 1 shows the discretized M × N array of point (or simple) sources of proposed conformal array geometry. These point sources are conventionally located at the center of the actual rectangular elements from where the pressure field is calculated, as shown in Figure 2. As clearly shown in Figure 2, the size of the elements in the M- and N-subarray parts of the structure are denoted by *L* and *W*, respectively. In Figure 2a, *R*_M_, *d*, and *α* are the radius of curvature, the pitch (inter-element spacing), and the angle of separation between two adjacent elements in the M-subarray part, respectively. Similarly, *R*_N_, *d*’, and *β* respectively denote the same quantities in the N-subarray part as shown in Figure 2b. *K*_w_ is the kerf (edge-to-edge spacing) on both sides of the array. The mathematical relationships between these geometrical parameters are presented in Equations (1)–(4). In these equations, *D*_M_ and *D*_N_ are the total widths of the array aperture in the M- and N-subarray parts, respectively.

(1) $$d=K_{w}+L=2R_{M}\sin\left(\alpha /2\right)$$

(2) $$D_{M}=\left(M-1\right)d+L$$

(3) $$d^{\prime }=K_{w}+W=2R_{N}\;\sin\left(\beta /2\right)$$

(4) $$D_{N}=\left(N-1\right)\;d^{\prime }+W$$

## 3. Determination of the Far-Field

The pressure from the array is calculated at the far-field, which is where the pressure amplitude varies inversely with the distance from the acoustic source [51]. As depicted in Figure 2, *m* = 1, 2, 3 … (M−1)/2, and *n* = 1, 2, 3 … (N−1)/2, respectively denote the positions of individual elements in the M- and N-subarray parts where both M and N are odd integers. Odd numbers of elements are relatively better than even numbers to mitigate the ripple [17]. The measurement angle as projected to the far-field point relative to the normal axis from each element in the M- and N-subarray parts is denoted as *θ* and *ϕ*, respectively. In the M-subarray, as shown in Figure 2a, *r* denotes the distance from the center element to the measurement point while the *r*_rm_ and *r*_lm_ terms denote these distances from the *m*th element on the right and left sections of the array, respectively. Equations (5)–(9) present the relationship between these quantities.

(5) $$r_{r1}=r+d\;\sin\left(\alpha /2-\theta \right)$$

(6) $$r_{r2}=r_{r1}+d\;\sin\left(3\alpha /2-\theta \right)$$

(7) $$r_{rm}=r_{r\left(m-1\right)}+d\;\sin\left[\left(2m-1\right)\;\alpha /2-\theta \right]$$

(8) $$r_{\mathrm{rm}}=r+2R_{M}\sin\left(\frac{\alpha }{2}-\theta \right)\sin\left(\alpha /2\right)\ldots +2R_{M}\sin\left[\left(2m-1\right)\frac{\alpha }{2}-\theta \right]\sin\left(\alpha /2\right)\;=r+R_{M}\cos\theta -R_{M}\cos\left(m\alpha -\theta \right)$$

(9) $$r_{\mathrm{lm}}=r+R_{M}\cos\theta -R_{M}\cos\left(m\alpha +\theta \right)$$

Similarly for the N-subarray in Figure 2b, where *r*’ denotes the far-field distance from the center element, the *r*’_rn_ and *r*’_ln_ representing the same far-field distance from the *n*^th^ element on the right and left section of the array, respectively, can also be obtained as presented in Equations (10) and (11).

(10) $${r^{\prime }}_{rn}=r^{\prime }+R_{N}\cos\emptyset -R_{N}\cos\left(n\beta -\emptyset \right)$$

(11) $${r^{\prime }}_{ln}=r^{\prime }+R_{N}\cos\emptyset -R_{N}\cos\left(n\beta +\emptyset \right)$$

## 4. Derivation of the Directivity Function

Fundamentally, Equation (12) presents the radiated sound pressure, *p*, from the simple source at any distance *R* [50]. In this equation, *A* is the pressure amplitude, *ω* is the angular frequency, *k* is the wave number, and *t* is the wave propagation time. Using this equation, the acoustic pressures, *P*_c_ from the central element as well as *P*_l_ and *P*_r_ from the left and right section of the M-subarray, respectively, are expressed in Equations (13)–(15).

(12) $$p=\frac{A}{R}e^{i\left(\omega t-kR\right)}$$

(13) $$P_{c}=\frac{A}{r}e^{i\left(\omega t-kr\right)}$$

(14) $$P_{l}=\overset{\frac{M-1}{2}}{\underset{m=1}{\sum }}\frac{A}{r_{lm}}e^{i\left(\omega t-kr_{lm}\right)}$$

(15) $$P_{r}=\overset{\frac{M-1}{2}}{\underset{m=1}{\sum }}\frac{A}{r_{rm}}e^{i\left(\omega t-kr_{rm}\right)}$$

At far-field, 1/*r*_rm_ = 1/*r*_lm_ ≈ 1/*r*. Hence, the sum of these pressures results in the total radiated pressure, *P*_M_, in Equation (16) and, subsequently, the corresponding directivity function, *H*_M_ (*θ*), of the M-subarray in Equation (17).

(16) $$P_{M}=\frac{A}{r}e^{i\left(\omega t-kr\right)}\left[1+\overset{\frac{M-1}{2}}{\underset{m=1}{\sum }}\left\{e^{-ikR_{M}\left(\cos\theta -\cos\left(m\alpha +\theta \right)\right)}+e^{-ikR_{M}\left(\cos\theta -\cos\left(m\alpha -\theta \right)\right)}\right\}\right]$$

(17) $$H_{M}\left(\theta \right)=\left[1+\overset{\frac{M-1}{2}}{\underset{m=1}{\sum }}\left\{e^{-ikR_{M}\left(\cos\theta -\cos\left(m\alpha +\theta \right)\right)}+e^{-ikR_{M}\left(\cos\theta -\cos\left(m\alpha -\theta \right)\right)}\right\}\right]$$

By a similar approach, the total radiated sound pressure, *P*_N_, and the corresponding directivity function, *H*_N_ (*ϕ*), for the N-subarray part can be expressed as Equations (18) and (19), respectively.

(18) $$P_{N}=\frac{A}{\;r\prime }e^{i\left(\omega t-k\;r\prime \right)}\left[1+\overset{\frac{N-1}{2}}{\underset{n=1}{\sum }}\left\{e^{-ikR_{N}\left(\cos\emptyset -\cos\left(n\beta +\emptyset \right)\right)}+e^{-ikR_{N}\left(\cos\emptyset -\cos\left(n\beta -\emptyset \right)\right)}\right\}\right]$$

(19) $$H_{N}\left(\emptyset \right)=\left[1+\overset{\frac{N-1}{2}}{\underset{n=1}{\sum }}\left\{e^{-ikR_{N}\left(\cos\emptyset -\cos\left(n\beta +\emptyset \right)\right)}+e^{-ikR_{N}\left(\cos\emptyset -\cos\left(n\beta -\emptyset \right)\right)}\right\}\right]$$

**Figure 3:**
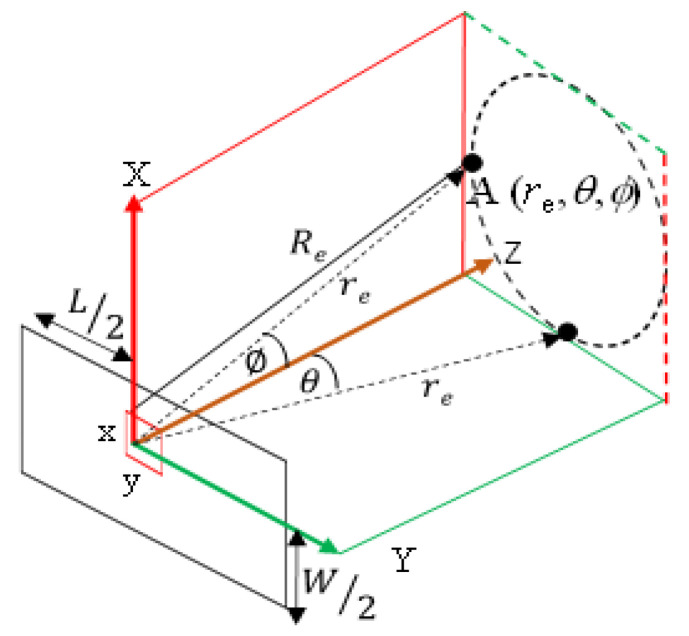
Analytical geometry for calculating the directional factor of the rectangular element.

Having assumed the active element as a simple source, the calculation of the actual directional factor of the proposed conformal array requires the determination of the directional factor, *H*_r_(*θ*, *ϕ*), of the actual rectangular element used in the array. To this end, Figure 3 shows the geometry for the derivation of *H*_r_(*θ*, *ϕ*), for a rectangular element of *L* × *W* dimension. 

In Figure 3, the distance from the center of the element to the measurement point A is *r*_e_ while *θ* and *ϕ* are zero in the orthogonal YZ and XZ planes, respectively. Subsequently, the distance *R*_e_ from any other point on an idealized small element xy can be expressed as in Equation (20). Based on this equation, the acoustic pressure of the rectangular element, *P*_e_, is derived as expressed in Equation (21) and finally resulting into *H*_r_(*θ*, *ϕ*) in Equation (22).

(20) $$R_{e}=r_{e}-x\sin\emptyset -y\sin\theta$$

(21) Pe=∫−W2W2∫−L2L2AReei(ωt−kRe)dxdy=Areei(ωt−kre)LWsin(kL2sinθ)kL2sinθ·sin(kW2sin∅)kW2sin∅

(22) $$H_{r}\left(\theta ,\emptyset \right)=\frac{\sin\left(\frac{kL}{2}\sin\theta \right)}{\frac{kL}{2}\sin\theta }\cdot \frac{\sin\left(\frac{kW}{2}\sin\emptyset \right)}{\frac{kW}{2}\sin\emptyset }$$

By normalizing the constituent subarray directional factors, *H*_M_(*θ*) and *H*_N_(*ϕ*), with their respective maximum values, and combining them with *H*_r_(*θ*, *ϕ*) using the product theorem [52], the overall directional factor of the whole conformal array, *H*(*θ*, *ϕ*), is obtained in Equation (23). Consequently, the beam pattern of the whole conformal array, *b*(*θ*, *ϕ*), is finally expressed in Equation (24).

(23) $$H\left(\theta ,\emptyset \right)=\left|\frac{H_{M}}{H_{M}{}_{\max}}\right|\cdot \left|\frac{H_{N}}{H_{N}{}_{\max}}\right|\cdot H_{r}\left(\theta ,\emptyset \right)$$

(24) b(θ,∅)=20log|H(θ,∅)|

## 5. The Working Frequency and the Maximum Array Aperture Size

Implementation of Equation (24) requires determining the operating frequency *f* and size of the array with respect to the relationship in Equation (25). In this equation, *c* = 1500 m/s is the acoustic wave speed in water while λ is the wavelength.

*k* = 2π*f*/*c* = 2π/*λ* (25)

The long-range detection is favored by the low-frequency range (1–100 kHz). Meanwhile, high ultrasonic frequency for acoustic imaging has also been placed between 100 kHz and 2 MHz [47]. Since resolution and frequency are directly related, 100 kHz is therefore selected as the working frequency of the proposed array. Being an interface between the low- and high-frequency signals, this frequency provides the highest possible resolution for low-frequency signals and an excellent long-penetration range of up to 1 km, and allows for centimeter size range of a small conformal array structure. 

Based on this frequency and the space limitation on the hosting surface of the mine hunter for the proposed array, the aperture width is restricted to a maximum of 33 cm, which also falls within the size range recommended for underwater acoustic imaging [47]. In addition, the pitch is maintained at less than a half wavelength while the kerf is also fixed at 0.5 mm to achieve the wider element size needed for the target high-power output. Its reduction below this value may facilitate mutual acoustic coupling between the elements or degrade the power output if higher. Application of these conditions to Equations (1)–(2) yields design constraints in Equations (26) and (27) where *a* = *L* or *W* and *b* = M or N, respectively, for the M- and N-subarray parts.

*a* < 7.0 (26)

*b* ≤ 330.5/(*a* + 0.5); (27)

## 6. Design of the Conformal Volumetric Array

**Figure 4:**
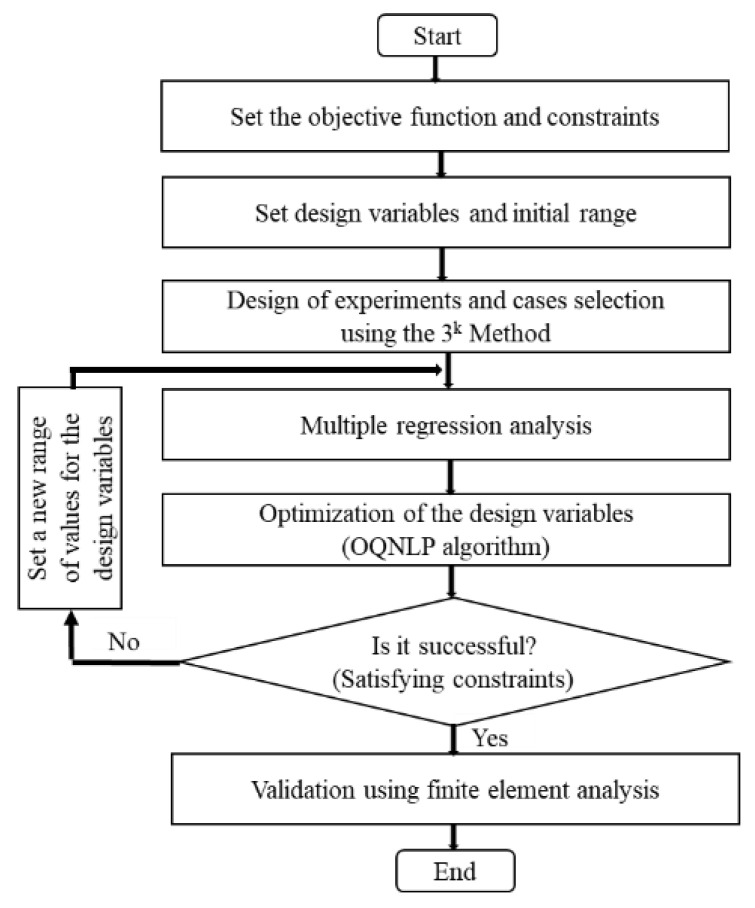
Flowchart for the optimization of the conformal array.

In this section, the final design of the structure was accomplished by optimization technique using the OptQuest Nonlinear Programming (OQNLP) algorithm [53,54]. The algorithm for the entire optimization procedure was implemented using a MATLAB program (version R2019a 9.6) and it is presented in the flowchart in Figure 4.

The effect of the geometrical variables on the performance characteristics was evaluated in Equation (24) using different values of M, *L*, and *R*_M_ for the M-array (at *ϕ* = 0) and N, *W*, and *R*_N_ in the case of the N-subarray part (at *θ* = 0). A MATLAB program for calculating the −3 dB beamwidth as well as the magnitude of the ripple formed within the main lobe was developed. Optimization technique by OQNLP algorithm requires a regression analysis and constraint equations. Regression analysis involves finding the best fitting function between the input and output variables using the least square method [55]. With a given value for the radius of curvature, both the size and number of elements were determined by the developed geometrical relations according to the conditions in Equations (26) and (27). 

For the M-subarray, *R*_M_ and *L* were varied at ± 0.1*R*_0_ and *L*_0_ ± 0.2 mm of their respective initial basic values, *R*_0_ and *L*_0_. They were then formulated into a 2 × J matrix of experimental models using the 3^k^ factorial design method [56]. The number of elements, M, was not included in the regression as a design variable. Each M-value was treated as a discrete constant value in each iterative process to preserve its value as an integer. With each M-value, *R*_M_ and *L* values in each J-column were then used to evaluate both the ripple and −3 dB beamwidth in the main lobe. 

Since the performance outputs indicate nonlinear relationships with the design variables, the quadratic polynomial functions in Equations (28) and (29) were derived for both output parameters Y_1_ and Y_2_ in terms of the design variables X_1_ and X_2_. The coefficients *B*_0_ … *B*_8_ and *C*_0_ … *C*_8_ were to be determined by the regression analysis. In this equation, Y_1_ = Ripple, Y_2_ = Beamwidth, X_1_ = *R*_M_/*R*_0_, and X_2_ = *L*/*L*_0_. The independent input variables, *R*_M_ and *L*, are divided by their basic values, respectively, because of the large difference between their physical values.

(28) $$Y_{1}=B_{0}+B_{1}X_{1}+B_{2}X_{2}+B_{3}X_{1}X_{2}+B_{4}X_{1}{}^{2}X_{2}+B_{5}X_{2}{}^{2}X_{1}+B_{6}X_{1}{}^{2}+B_{7}X_{2}{}^{2}+B_{8}X_{1}{}^{2}X_{2}{}^{2}$$

(29) $$Y_{2}\;=C_{0}+C_{1}X_{1}+C_{2}X_{2}+C_{3}X_{1}X_{2}+C_{4}X_{1}{}^{2}X_{2}+C_{5}X_{2}{}^{2}X_{1}+C_{6}X_{1}{}^{2}+C_{7}X_{2}{}^{2}+C_{8}X_{1}{}^{2}X_{2}{}^{2}$$

The coefficients in Equations (28) and (29) were evaluated resulting in a perfect fitting function in Equations (30) and (31) having a regression coefficient of unity.

(30) $$Y_{1}=19.7290-70.5376X_{1}{}^{2}-18.4869X_{2}{}^{2}+71.8826X_{1}{}^{2}X_{2}$$

(31) $$Y_{2}\;=\left(-1.3573+1.2303X_{1}{}^{2}+1.485X_{2}{}^{2}-1.2828X_{1}{}^{2}X_{2}\right)\times 10^{3}$$

where $X_{1}\;$= *R*_M_/202.8, $X_{2}$ = *L*/5.2.

Based on this regression analysis result, optimization was conducted by the OQNLP algorithm to achieve the objective in Equation (32). The design target was to minimize the ripple and achieve a −3 dB beamwidth of 85° in the M-subarray part. Hence, the objective function and the constraint for the optimization were set as Equation (32) where *y*_l_ and *y*_u_ are 85° and 90°, respectively. This process was iterated for different trial values of the design variables until this equation was satisfied according to Figure 4. This was eventually achieved at a point when M = 55 where the penultimate range of design variables was as shown in Table 1.

(32) {Objective : Minimize Y1 Constraint: yl≤Y2≤yu

**Figure 5:**
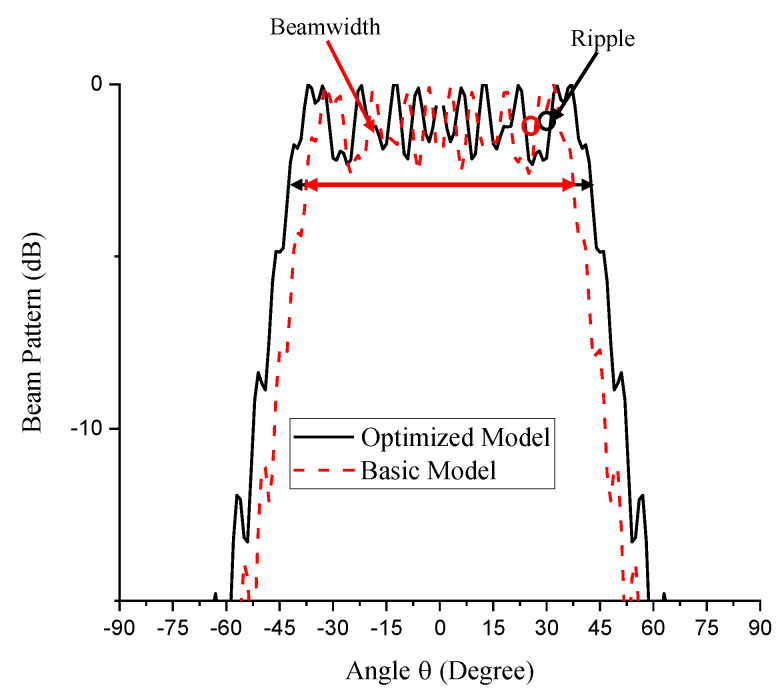
The beam pattern of the M-subarray part of the optimized conformal structure compared with the basic model.

The final result was obtained as shown in Table 2. The result in Table 2 clearly shows that the ripple has been minimized by 0.3 dB while the beamwidth has been widened by 10° to the desired 85.1°. Figure 5 shows the comparison between the beam patterns of the initial and optimized models. By this result, the conformal array has the final aperture size of 31.3 cm and the pitch of 5.7 mm (0.38λ). This result agrees with the finding that a minimal ripple is obtained at reduced pitch *d* = 0.4λ [49]. The maximum aperture width also falls in the smaller size region of the recommended range of 10 cm–1 m indicating good economic consideration [47].

**Figure 6:**
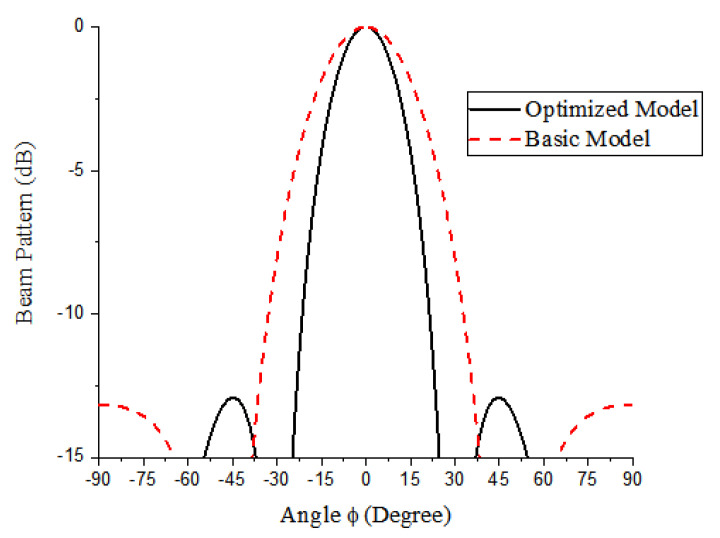
The beam pattern of the N-subarray part of the optimized conformal structure compared with the basic model.

Having completed the design of the M-subarray, the similar procedure was carried out for the N-subarray using N, *R*_N_, and *W*. Now, X_1_ and X_2_ in Equations (28) and (29) are *R*_N_/*R*_0_ and *W*/*W*_0_, respectively. Due to the −3 dB beamwidth target of 25°, *y_l_* and *y*_u_ in Equation (32) for the N-subarray part are 25° and 26°, respectively. The penultimate range of the design variables for this part is presented in Table 3. The regression analysis yielded a perfect fitting function having a regression coefficient of unity presented in Equations (33) and (34). In Equation (33), Y_1_ = 0 because there is no ripple in the N-subarray part due to the small number of elements, N = 5 producing the target beamwidth as shown in the final optimization result in Table 4. Consequently, the pitch and aperture width on this part are 6.1 mm (0.4λ) and 3 cm, respectively. Figure 6 shows the comparison between the beam patterns of the initial and optimized models.

(33) $$Y_{1}=0$$

(34) $$Y_{2}\;=132.5495-0.2075X_{1}-140.5653X_{2}+0.2246X_{1}X_{2}-0.0940X_{1}{}^{2}X_{2}\;\;\;\;\;\;\;\;\;\;-0.3597X_{2}{}^{2}X_{1}+0.0809X_{1}{}^{2}+46.2380X_{2}{}^{2}+0.1396X_{1}{}^{2}X_{2}{}^{2}$$

where $X_{1}\;$= *R*_N_/120, $X_{2}\;$= *W*/3.6.

## 7. Validation of the Design

Finite element analysis (FEA) has been described as the best way to obtain quantitative data for an acoustic array design [52]. Its reliability to evaluate the integrity of theoretical designs in academic and commercial applications has been emphasized [57,58]. Consequently, a commercial FEA software, Pzflex^®^ (version 1.21.7.0), is employed to validate the design as well as evaluate the TVR level of the conformal array with respect to the target minimum of 147 dB. PZT-5H was selected as the active piezoceramic element, as adopted from [52], while other structural components are presented in Table 5. This FEA model was constructed in consideration of the operational factors in the practical underwater environment.

**Figure 7:**
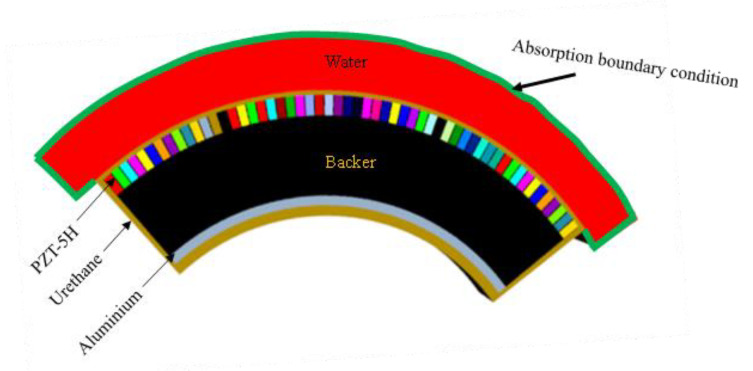
The cross-section of the FEA model of the 5 × 55 conformal array.

The cross-section of the FEA model of the whole array structure is shown in Figure 7. Each of the piezoceramic elements was excited with an impulse signal of 1 volt as a source of electrical energy. The ceramic element was placed between the layers of urethane and the backer. To provide an additional layer of support to the structure, aluminum was placed behind the backer. A urethane covering was applied to the entire geometrical matrix to serve both as an acoustic window for impedance matching as well as waterproofing. To simulate the transducer’s real-world working environment, water was modeled on the front surface of the array as the acoustic radiation medium. An absorption boundary condition was applied around the water domain to prevent reflection of acoustic waves. The curvature and the dimension of the radiating surface were exactly as previously obtained in the theoretical design for the rectangular element. Since the transducer vibrates in the thickness mode, the thickness of the transducer was controlled by the operating frequency. 

**Figure 8:**
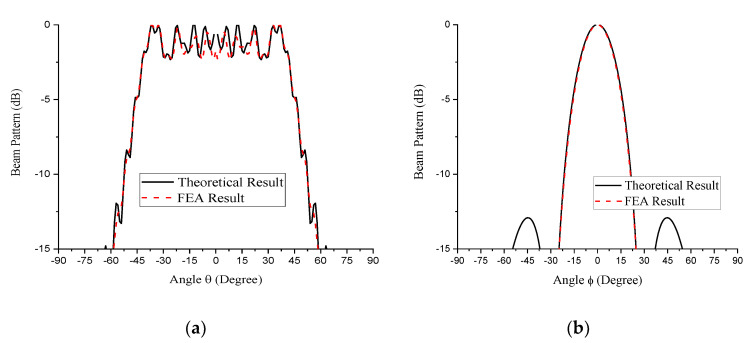
Two-dimensional theoretical beam pattern of the 5 × 55 conformal array compared with those from the FEA: (**a**) M-subarray part; (**b**) N-subarray part.

**Figure 9:**
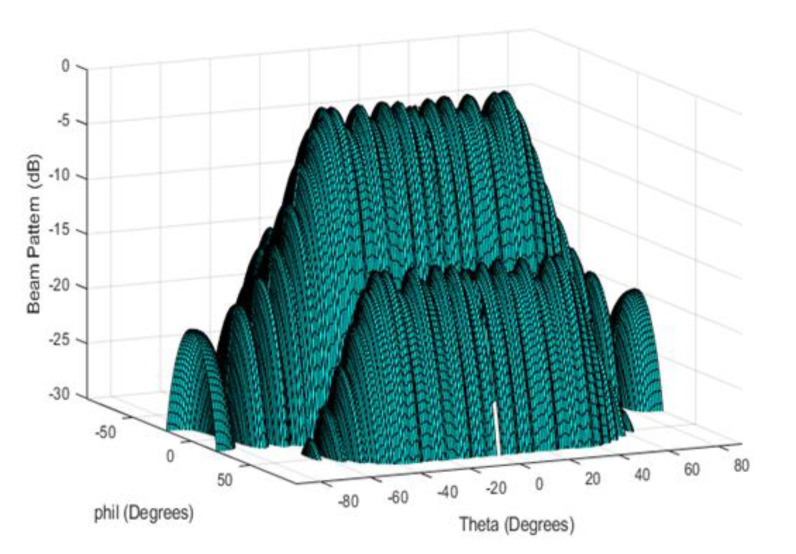
Three-dimensional beam pattern of the 5 × 55 conformal array.

The outcome of the FEA was compared with the optimized analytical results for the M- and N-subarrays presented in Figure 5 and Figure 6, respectively, as shown in Figure 8. The excellent agreements especially at the main lobe of the beam patterns provides sound proof to the validity of our novel theoretical design approach. Additionally, the three-dimensional plot of the beam is shown in Figure 9 while the quantitative difference between the two spectra is presented in Table 6. 

**Figure 10:**
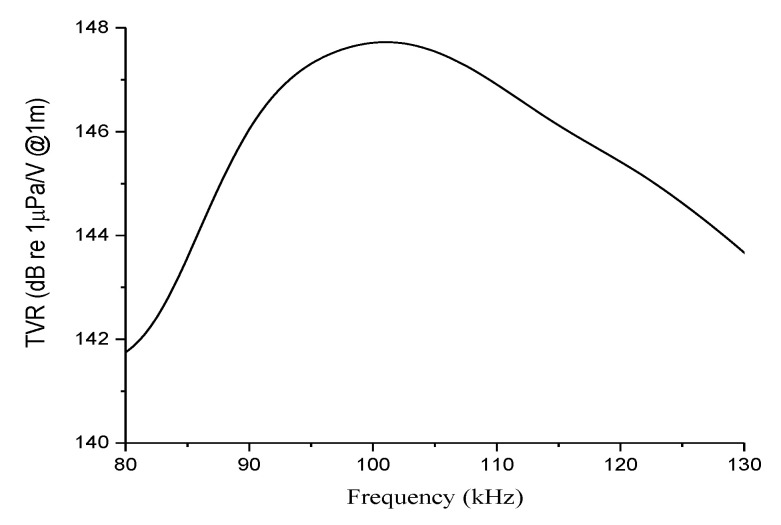
TVR spectrum of the 5 × 55 conformal array.

Finally, Figure 10 shows the resulting TVR spectrum with a peak level of 147.7 dB, further satisfying the design target of the proposed conformal volumetric array.

## 8. Conclusions

This study presents a novel, simple, and systematic design technique for a 5 × 55 conformal volumetric array for sonar detection to solve the maritime security challenge posed by underwater mines. The design was conducted using the maximum low-frequency of 100 kHz, beamwidth of 85° at a controlled aperture size, and a transmitting voltage response of 147.7 dB to break even from the major performance issues in underwater acoustic imaging for sonar detection. The combination of odd-number elements and optimization technique were used to determine the least possible ripple magnitude. It is spectacular that the minimum ripple was obtained at the effective pitch of 0.38λ in exact agreement with the existing theory. The maximum array size of 31.3 cm also falls within the small category of the acceptable range indicating the economic value of the design. Nevertheless, the integrity of our approach was again validated using the finite element analysis and an excellent compliance was attained. This method is not only computationally accurate but also uniquely simpler, faster, and more natural. It has been used to design a structure with a specific curvature and frequency in the present work as a sample case. However, the design equation was developed as a universal model for any arbitrary frequency depending on the interest of the designer. It is a versatile template that is adaptable to other curvatures for different performance specifications. Consequently, it is suitable as a good reference for future development of conformal volumetric arrays for underwater mine detection.

## Figures and Tables

**Table 1:** Variation range of the design variables for the M-subarray part.

Design Variable	Lower Bound	Basic	Upper Bound
Radius of Curvature, *R*_M_ (mm)	182.5	202.8	223.1
Length of Element, *L* (mm)	5.0	5.2	5.4

**Table 2:** Comparison between basic and optimized models for the M-subarray part.

Model	Geometrical Parameters			Performance Parameters	
	M	*R*_M_ (mm)	*L* (mm)	Ripple (dB)	Beamwidth (°)
Basic	55	202.8	5.2	2.6	75.1
Optimized	55	182.5	5.2	2.3	85.1

**Table 3:** Variation range of design variables for the N-subarray part.

Design Variables	Lower Bound	Basic	Upper Bound
Radius of Curvature, *R*_N_ (mm)	80	120	160
Width of Element, *W* (mm)	1.6	3.6	5.6

**Table 4:** Comparison between basic and optimized models for the N-subarray part.

Model	Geometrical Parameters			Performance Parameters	
	N	*R*_N_ (mm)	*W* (mm)	Ripple (dB)	Beamwidth (°)
Basic	5	120	3.6	0	38.0
Optimized	5	150	5.6	0	25.3

**Table 5:** Material properties of constituent components of the conformal array.

Material	Density (kg/m^3^)	Longitudinal Velocity (m/s)	Shear Velocity (m/s)
Urethane/Kerf	1065	1284	252
Backer	1712	1815	0
Aluminum	2700	6149	3097
Water	1000	1500	0

**Table 6:** Quantitative comparison between the theoretical and FEA results.

	M-Array Part		N-Array Part	
Measured Parameters	Theoretical	FEA	Theoretical	FEA
−3 dB Beamwidth (°)	85.1	85.2	25.3	24.5
Ripple level (dB)	2.3	2.3	0	0

## Data Availability

Not applicable.
